# (2,4-Dipropoxyphen­yl)boronic acid

**DOI:** 10.1107/S1600536811049737

**Published:** 2011-11-30

**Authors:** Marek Dąbrowski, Krzysztof Durka, Sergiusz Luliński, Janusz Serwatowski

**Affiliations:** a Physical Chemistry Department, Faculty of Chemistry, Warsaw University of Technology, Noakowskiego 3, 00-664 Warsaw, Poland

## Abstract

In the crystal, the title compound, C_12_H_19_BO_4_, exists as a centrosymmetric O—H⋯O hydrogen-bonded dimer. Dimers are linked *via* C—H⋯O hydrogen bonds, generating an infinite zigzag chain oriented parallel to [1
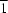
1]. The chains are assembled, giving sheets aligned parallel to (21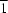) and inter­connected by weak C—H⋯π inter­actions, producing a three-dimensional network.

## Related literature

For the structural characterization of related *ortho*-alk­oxy aryl­boronic acids, see: Dąbrowski *et al.* (2008 ▶, 2009 ▶); Yang *et al.* (2005 ▶).
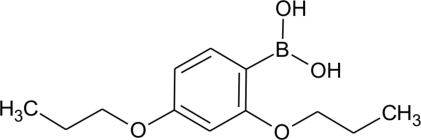


## Experimental

### 

#### Crystal data

C_12_H_19_BO_4_
*M* *_r_* = 238.08
Triclinic, 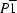

*a* = 7.9630 (9) Å
*b* = 8.8014 (12) Å
*c* = 9.3182 (13) Å
α = 101.585 (11)°
β = 91.924 (10)°
γ = 90.826 (10)°
*V* = 639.26 (15) Å^3^
*Z* = 2
Mo *K*α radiation
μ = 0.09 mm^−1^
*T* = 100 K
0.15 × 0.12 × 0.10 mm

#### Data collection

Bruker APEXII diffractometer
Absorption correction: multi-scan (*SORTAV*; Blessing, 1995 ▶) *T*_min_ = 0.986, *T*_max_ = 0.992
12243 measured reflections
2950 independent reflections
1981 reflections with *I* > 2σ(*I*)
*R*_int_ = 0.024

#### Refinement

*R*[*F* ^2^ > 2σ(*F* ^2^)] = 0.033
*wR*(*F* ^2^) = 0.081
*S* = 0.90
2950 reflections
154 parameters
H-atom parameters constrained
Δρ_max_ = 0.35 e Å^−3^
Δρ_min_ = −0.19 e Å^−3^

### 

Data collection: *APEX2* (Bruker, 2010 ▶); cell refinement: *SAINT* (Bruker, 2010 ▶); data reduction: *SAINT*; program(s) used to solve structure: *SHELXS97* (Sheldrick, 2008 ▶); program(s) used to refine structure: *SHELXS97* (Sheldrick, 2008 ▶); molecular graphics: *DIAMOND* (Brandenburg, 2005 ▶); software used to prepare material for publication: *PLATON* (Spek, 2009 ▶).

## Supplementary Material

Crystal structure: contains datablock(s) global, I. DOI: 10.1107/S1600536811049737/zj2041sup1.cif

Structure factors: contains datablock(s) I. DOI: 10.1107/S1600536811049737/zj2041Isup2.hkl

Supplementary material file. DOI: 10.1107/S1600536811049737/zj2041Isup3.cml

Additional supplementary materials:  crystallographic information; 3D view; checkCIF report

## Figures and Tables

**Table 1:** Hydrogen-bond geometry (Å, °)

*D*—H⋯*A*	*D*—H	H⋯*A*	*D*⋯*A*	*D*—H⋯*A*
O1—H1⋯O2^i^	0.84	1.96	2.794 (1)	176
O2—H2⋯O3	0.84	1.95	2.672 (1)	144
C5—H5⋯O4^ii^	0.95	2.50	3.445 (1)	175
C10—H10*B*⋯O1^iii^	0.99	2.84	3.78 (1)	158
C8—H8*B*⋯*Cg*1^iv^	0.99	2.83	3.671 (1)	143

Symmetry codes: (i) 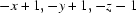; (ii) 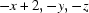; (iii) 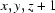; (iv) 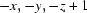.

**Fig. 1.**
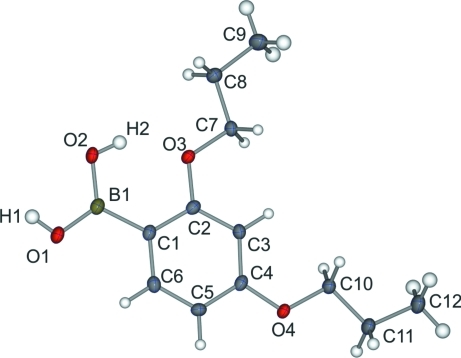
The molecular structure of the title compound (I) with the atom numbering scheme. Displacement ellipsoids for non-H atoms are drawn at the 50% probability level.

**Fig. 2.**
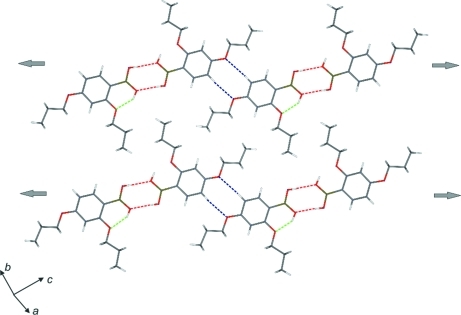
Formation of two-dimensional layer constructed from one-dimensional chains, which are generated through O—H···O and C—H···O interactions (red and blue colours, respectively).

**Fig. 3.**
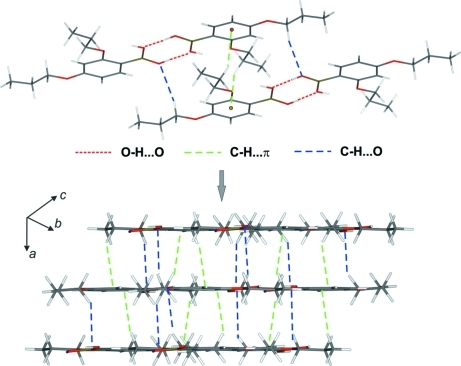
The three-dimensional supramolecular structure of (I). Intermolecular C—H···O and C—H···π interactions formed between two-dimensional layers are depicted as blue and green lines, respectively.

## supplementary crystallographic information

### Comment

The ability of arylboronic acids to form supramolecular assemblies due to intermolecular hydrogen bonding is well known. Our interest has focused on *ortho*-alkoxy derivatives and the influence of various factors (including the number and length of the alkoxy group) on their structural behaviour.

The molecular structure of (I) shows the boronic groups possesses an *exo*-*endo* conformation. The entire molecule including both propoxy groups remains essentially planar. The *endo*-oriented OH group is engaged in an intramolecular O—H···O hydrogen bond (Table 1) with the 2-propoxy O atom, resulting in the formation of a six-membered ring. This motif is generally typical for structures of all *ortho*-alkoxyarylboronic acids (Yang *et al.*, 2005; Dąbrowski *et al.*, 2008; Luliński, 2008).

Centrosymmetric O—H···O hydrogen-bonded dimers of (I) are linked by weaker C—H···O hydrogen bonds connecting the H5 atom attached to aromatic ring with the O atom of the 4-propoxy group in the adjacent molecule. Thus, another centrosymmetric dimeric motif can be distinguished. These two alternating dimeric motifs generate a zig-zag chain which runs along the [111] direction. Adjacent chains are ordered due to van der Waals interactions of propoxy groups which leads to the formation of a 2D layer aligned parallel to the (211) plane. The supramolecular architecture extends further due to weak C—H···O contacts between α-methylene units of 4-propoxy groups and one of O atoms of the boronic group. Finally, C—H···π interactions occur between the β-methylene units of the 2-propoxy group and the aromatic ring of a molecule in the adjacent layer. As a result, a three-dimensional network is formed.

### Experimental

The title compound was received from Aldrich. Crystals suitable for single-crystal X-ray diffraction analysis were grown by slow evaporation of a solution of the acid (0.2 g) in acetone/water (10 ml, 1:1).

### Refinement

All hydrogen atoms were placed in calculated positions with C—H distance of 0.95Å (phenyl), 0.98Å (methyl), 0.99Å (methylene) and O—H distance of 0.84 Å.They were visible in difference maps and they were included in the refinement in riding-motion approximation with *U*_iso_(phenyl H)=1.2*U*_eq_(C), *U*_iso_(methyl H)=1.5*U*_eq_(C) and *U*_iso_(OH H)=1.5*U*_eq_(O).

### Crystal data

**Table:**

C_12_H_19_BO_4_	*Z* = 2
*M**_r_* = 238.08	*F*(000) = 256
Triclinic, *P*1	*D*_x_ = 1.237 Mg m^−^^3^
Hall symbol: -P 1	Mo *K*α radiation, λ = 0.71073 Å
*a* = 7.9630 (9) Å	Cell parameters from 1540 reflections
*b* = 8.8014 (12) Å	θ = 2.7–28.4°
*c* = 9.3182 (13) Å	µ = 0.09 mm^−^^1^
α = 101.585 (11)°	*T* = 100 K
β = 91.924 (10)°	Unshaped, colourless
γ = 90.826 (10)°	0.15 × 0.12 × 0.10 mm
*V* = 639.26 (15) Å^3^	

### Data collection

**Table:**

Bruker APEXII diffractometer	2950 independent reflections
Radiation source: TXS rotating anode	1981 reflections with *I* > 2σ(*I*)
multi-layer optics	*R*_int_ = 0.024
ω scans	θ_max_ = 27.5°, θ_min_ = 2.9°
Absorption correction: multi-scan (*SORTAV*; Blessing, 1995)	*h* = −10→10
*T*_min_ = 0.986, *T*_max_ = 0.992	*k* = −11→11
12243 measured reflections	*l* = −12→12

### Refinement

**Table:**

Refinement on *F*^2^	Primary atom site location: structure-invariant direct methods
Least-squares matrix: full	Secondary atom site location: difference Fourier map
*R*[*F*^2^ > 2σ(*F*^2^)] = 0.033	Hydrogen site location: inferred from neighbouring sites
*wR*(*F*^2^) = 0.081	H-atom parameters constrained
*S* = 0.90	*w* = 1/[σ^2^(*F*_o_^2^) + (0.0487*P*)^2^] where *P* = (*F*_o_^2^ + 2*F*_c_^2^)/3
2950 reflections	(Δ/σ)_max_ < 0.001
154 parameters	Δρ_max_ = 0.35 e Å^−^^3^
0 restraints	Δρ_min_ = −0.19 e Å^−^^3^

### Special details

**Table:**

Geometry. Bond distances, angles *etc*. have been calculated using the rounded fractional coordinates. All su's are estimated from the variances of the (full) variance-covariance matrix. The cell e.s.d.'s are taken into account in the estimation of distances, angles and torsion angles
Refinement. Refinement of *F*^2^ against ALL reflections. The weighted *R*-factor *wR* and goodness of fit *S* are based on *F*^2^, conventional *R*-factors *R* are based on *F*, with *F* set to zero for negative *F*^2^. The threshold expression of *F*^2^ > σ(*F*^2^) is used only for calculating *R*-factors(gt) *etc*. and is not relevant to the choice of reflections for refinement. *R*-factors based on *F*^2^ are statistically about twice as large as those based on *F*, and *R*- factors based on ALL data will be even larger.

### Fractional atomic coordinates and isotropic or equivalent isotropic displacement parameters (Å^2^)

**Table:**

	*x*	*y*	*z*	*U*_iso_*/*U*_eq_	
O1	0.62433 (10)	0.32104 (9)	−0.46474 (8)	0.0260 (3)	
O2	0.54695 (10)	0.55834 (9)	−0.31800 (8)	0.0243 (3)	
O3	0.67310 (10)	0.60724 (8)	−0.04282 (8)	0.0209 (2)	
O4	0.96656 (9)	0.20013 (9)	0.13916 (8)	0.0211 (2)	
C1	0.72028 (13)	0.36735 (12)	−0.19957 (11)	0.0169 (3)	
C2	0.73803 (13)	0.46070 (12)	−0.05836 (11)	0.0174 (3)	
C3	0.81690 (13)	0.40958 (12)	0.05908 (11)	0.0176 (3)	
C4	0.88240 (13)	0.26147 (13)	0.03411 (11)	0.0173 (3)	
C5	0.86780 (13)	0.16440 (12)	−0.10371 (11)	0.0181 (3)	
C6	0.78780 (13)	0.21836 (13)	−0.21711 (11)	0.0187 (3)	
C7	0.69286 (13)	0.71279 (12)	0.09706 (11)	0.0177 (3)	
C8	0.61739 (14)	0.86468 (12)	0.08068 (12)	0.0215 (3)	
C9	0.62471 (15)	0.98129 (13)	0.22669 (12)	0.0282 (4)	
C10	0.99022 (14)	0.29466 (12)	0.28420 (11)	0.0192 (3)	
C11	1.09520 (15)	0.20257 (13)	0.37344 (11)	0.0227 (3)	
C12	1.13065 (15)	0.29436 (14)	0.52911 (12)	0.0274 (4)	
B1	0.62724 (15)	0.41844 (14)	−0.33306 (13)	0.0179 (3)	
H1	0.57125	0.36132	−0.52650	0.0390*	
H2	0.56001	0.60749	−0.23113	0.0363*	
H3	0.82554	0.47458	0.15376	0.0212*	
H5	0.91199	0.06295	−0.11955	0.0218*	
H6	0.77807	0.15181	−0.31097	0.0224*	
H7A	0.63476	0.67028	0.17313	0.0212*	
H7B	0.81346	0.72820	0.12665	0.0212*	
H8A	0.67939	0.90801	0.00721	0.0258*	
H8B	0.49893	0.84662	0.04476	0.0258*	
H9A	0.57467	1.07872	0.21307	0.0423*	
H9B	0.56207	0.93909	0.29913	0.0423*	
H9C	0.74208	1.00076	0.26141	0.0423*	
H10A	1.04869	0.39351	0.27963	0.0231*	
H10B	0.88032	0.31853	0.32916	0.0231*	
H11A	1.03500	0.10437	0.37739	0.0272*	
H11B	1.20288	0.17630	0.32526	0.0272*	
H12A	1.19865	0.23225	0.58440	0.0411*	
H12B	1.19187	0.39080	0.52538	0.0411*	
H12C	1.02418	0.31895	0.57745	0.0411*	

### Atomic displacement parameters (Å^2^)

**Table:**

	*U*^11^	*U*^22^	*U*^33^	*U*^12^	*U*^13^	*U*^23^
O1	0.0343 (5)	0.0274 (5)	0.0162 (4)	0.0093 (4)	−0.0060 (3)	0.0047 (3)
O2	0.0343 (5)	0.0246 (4)	0.0133 (4)	0.0074 (3)	−0.0056 (3)	0.0031 (3)
O3	0.0294 (4)	0.0171 (4)	0.0156 (4)	0.0066 (3)	−0.0050 (3)	0.0026 (3)
O4	0.0283 (4)	0.0201 (4)	0.0146 (4)	0.0076 (3)	−0.0057 (3)	0.0031 (3)
C1	0.0165 (5)	0.0199 (5)	0.0150 (5)	0.0004 (4)	−0.0009 (4)	0.0056 (4)
C2	0.0173 (5)	0.0176 (5)	0.0182 (5)	0.0023 (4)	−0.0004 (4)	0.0061 (4)
C3	0.0204 (5)	0.0186 (5)	0.0132 (5)	0.0019 (4)	−0.0014 (4)	0.0017 (4)
C4	0.0163 (5)	0.0208 (5)	0.0160 (5)	0.0011 (4)	−0.0016 (4)	0.0072 (4)
C5	0.0200 (6)	0.0149 (5)	0.0194 (6)	0.0038 (4)	0.0004 (4)	0.0031 (4)
C6	0.0193 (5)	0.0221 (6)	0.0142 (5)	0.0000 (4)	−0.0009 (4)	0.0027 (4)
C7	0.0200 (5)	0.0190 (6)	0.0134 (5)	0.0013 (4)	−0.0021 (4)	0.0021 (4)
C8	0.0257 (6)	0.0189 (6)	0.0201 (6)	0.0038 (5)	−0.0012 (4)	0.0048 (5)
C9	0.0351 (7)	0.0210 (6)	0.0269 (6)	0.0047 (5)	−0.0034 (5)	0.0016 (5)
C10	0.0240 (6)	0.0182 (5)	0.0149 (5)	0.0032 (4)	−0.0030 (4)	0.0023 (4)
C11	0.0288 (6)	0.0225 (6)	0.0167 (5)	0.0046 (5)	−0.0049 (4)	0.0041 (5)
C12	0.0357 (7)	0.0281 (7)	0.0182 (6)	0.0041 (5)	−0.0063 (5)	0.0051 (5)
B1	0.0167 (6)	0.0206 (6)	0.0175 (6)	0.0000 (5)	−0.0010 (5)	0.0067 (5)

### Geometric parameters (Å, °)

**Table:**

O1—B1	1.3476 (14)	C11—C12	1.5270 (15)
O2—B1	1.3798 (15)	C3—H3	0.9500
O3—C2	1.3788 (13)	C5—H5	0.9500
O3—C7	1.4431 (13)	C6—H6	0.9500
O4—C4	1.3702 (13)	C7—H7A	0.9900
O4—C10	1.4426 (13)	C7—H7B	0.9900
O1—H1	0.8400	C8—H8A	0.9900
O2—H2	0.8400	C8—H8B	0.9900
C1—C2	1.4058 (14)	C9—H9A	0.9800
C1—C6	1.4055 (16)	C9—H9B	0.9800
C1—B1	1.5719 (16)	C9—H9C	0.9800
C2—C3	1.3979 (15)	C10—H10A	0.9900
C3—C4	1.3898 (16)	C10—H10B	0.9900
C4—C5	1.3925 (15)	C11—H11A	0.9900
C5—C6	1.3831 (15)	C11—H11B	0.9900
C7—C8	1.5068 (15)	C12—H12A	0.9800
C8—C9	1.5297 (16)	C12—H12B	0.9800
C10—C11	1.5135 (16)	C12—H12C	0.9800
			
O1···O2^i^	2.7938 (12)	H3···C10	2.5400
O2···O3	2.6722 (11)	H3···H7A	2.3000
O2···O1^i^	2.7938 (12)	H3···H7B	2.3000
O3···O2	2.6722 (11)	H3···H10A	2.3000
O1···H10B^ii^	2.8400	H3···H10B	2.3700
O1···H6	2.5600	H5···O4^iv^	2.5000
O2···H1^i^	1.9600	H5···C11^iv^	2.9700
O3···H2	1.9500	H6···O1	2.5600
O4···H9C^iii^	2.9000	H7A···C3	2.7800
O4···H5^iv^	2.5000	H7A···H3	2.3000
C1···C7^v^	3.5564 (15)	H7A···H9B	2.5100
C7···C1^v^	3.5564 (15)	H7A···C1^v^	2.8700
C8···C8^vi^	3.5788 (16)	H7A···B1^v^	2.8000
C1···H10A^vii^	3.0000	H7A···H12A^viii^	2.5700
C1···H7A^v^	2.8700	H7B···C3	2.7500
C2···H2	2.6500	H7B···H3	2.3000
C3···H7B	2.7500	H7B···H9C	2.5600
C3···H10B	2.8200	H7B···C4^vii^	2.9000
C3···H7A	2.7800	H7B···C5^vii^	2.7300
C3···H10A	2.7400	H8A···C5^ix^	3.0600
C4···H7B^vii^	2.9000	H8B···C5^v^	2.9900
C5···H7B^vii^	2.7300	H8B···C6^v^	2.9500
C5···H8B^v^	2.9900	H9B···H7A	2.5100
C5···H8A^iii^	3.0600	H9B···C6^v^	3.1000
C6···H9B^v^	3.1000	H9C···O4^ix^	2.9000
C6···H12C^ii^	2.9800	H9C···H7B	2.5600
C6···H8B^v^	2.9500	H10A···C3	2.7400
C7···H12A^viii^	3.0000	H10A···H3	2.3000
C7···H3	2.5000	H10A···H12B	2.5300
C10···H3	2.5400	H10A···C1^vii^	3.0000
C11···H5^iv^	2.9700	H10A···B1^vii^	3.0200
B1···H1^i^	2.9900	H10B···O1^x^	2.8400
B1···H7A^v^	2.8000	H10B···C3	2.8200
B1···H10A^vii^	3.0200	H10B···H3	2.3700
H1···O2^i^	1.9600	H10B···H12C	2.5500
H1···B1^i^	2.9900	H12A···C7^viii^	3.0000
H1···H2^i^	2.5200	H12A···H7A^viii^	2.5700
H2···O3	1.9500	H12B···H10A	2.5300
H2···C2	2.6500	H12C···C6^x^	2.9800
H2···H1^i^	2.5200	H12C···H10B	2.5500
H3···C7	2.5000		
			
C2—O3—C7	119.07 (8)	C7—C8—H8B	109.00
C4—O4—C10	118.39 (8)	C7—C8—H8A	109.00
B1—O1—H1	109.00	C9—C8—H8A	109.00
B1—O2—H2	109.00	C9—C8—H8B	109.00
C2—C1—C6	116.11 (9)	H8A—C8—H8B	108.00
C2—C1—B1	124.11 (10)	C8—C9—H9B	109.00
C6—C1—B1	119.76 (9)	C8—C9—H9C	109.00
O3—C2—C1	115.62 (9)	H9A—C9—H9B	109.00
C1—C2—C3	122.57 (10)	C8—C9—H9A	109.00
O3—C2—C3	121.81 (9)	H9A—C9—H9C	109.00
C2—C3—C4	118.46 (9)	H9B—C9—H9C	109.00
O4—C4—C5	114.96 (10)	O4—C10—H10A	110.00
O4—C4—C3	123.87 (9)	H10A—C10—H10B	109.00
C3—C4—C5	121.17 (10)	C11—C10—H10B	110.00
C4—C5—C6	118.80 (10)	O4—C10—H10B	110.00
C1—C6—C5	122.88 (10)	C11—C10—H10A	110.00
O3—C7—C8	107.64 (8)	C10—C11—H11B	109.00
C7—C8—C9	111.13 (9)	C10—C11—H11A	109.00
O4—C10—C11	106.96 (8)	C12—C11—H11A	109.00
C10—C11—C12	111.14 (9)	C12—C11—H11B	109.00
C2—C3—H3	121.00	H11A—C11—H11B	108.00
C4—C3—H3	121.00	C11—C12—H12B	109.00
C6—C5—H5	121.00	C11—C12—H12C	109.00
C4—C5—H5	121.00	H12A—C12—H12B	109.00
C1—C6—H6	119.00	C11—C12—H12A	109.00
C5—C6—H6	119.00	H12A—C12—H12C	109.00
O3—C7—H7A	110.00	H12B—C12—H12C	109.00
O3—C7—H7B	110.00	O2—B1—C1	121.77 (10)
H7A—C7—H7B	108.00	O1—B1—O2	119.62 (10)
C8—C7—H7B	110.00	O1—B1—C1	118.60 (10)
C8—C7—H7A	110.00		
			
C7—O3—C2—C1	−177.25 (9)	C2—C1—B1—O1	177.34 (10)
C7—O3—C2—C3	2.44 (14)	B1—C1—C2—O3	−2.44 (15)
C2—O3—C7—C8	177.61 (9)	C6—C1—B1—O1	−4.42 (15)
C10—O4—C4—C5	178.70 (9)	O3—C2—C3—C4	−178.64 (10)
C10—O4—C4—C3	−0.52 (15)	C1—C2—C3—C4	1.03 (16)
C4—O4—C10—C11	−176.48 (9)	C2—C3—C4—C5	−1.12 (16)
C6—C1—C2—O3	179.27 (9)	C2—C3—C4—O4	178.05 (10)
C2—C1—B1—O2	−3.72 (17)	C3—C4—C5—C6	0.62 (16)
B1—C1—C6—C5	−178.48 (10)	O4—C4—C5—C6	−178.62 (9)
C6—C1—B1—O2	174.52 (10)	C4—C5—C6—C1	0.00 (17)
B1—C1—C2—C3	177.87 (10)	O3—C7—C8—C9	177.03 (9)
C2—C1—C6—C5	−0.11 (16)	O4—C10—C11—C12	178.86 (9)
C6—C1—C2—C3	−0.42 (15)		

Symmetry codes: (i) −*x*+1, −*y*+1, −*z*−1; (ii) *x*, *y*, *z*−1; (iii) *x*, *y*−1, *z*; (iv) −*x*+2, −*y*, −*z*; (v) −*x*+1, −*y*+1, −*z*; (vi) −*x*+1, −*y*+2, −*z*; (vii) −*x*+2, −*y*+1, −*z*; (viii) −*x*+2, −*y*+1, −*z*+1; (ix) *x*, *y*+1, *z*; (x) *x*, *y*, *z*+1.

### Hydrogen-bond geometry (Å, °)

**Table:**

*D*—H···*A*	*D*—H	H···*A*	*D*···*A*	*D*—H···*A*
O1—H1···O2^i^	0.840	1.960	2.794 (1)	176.0
O2—H2···O3	0.840	1.950	2.672 (1)	144.0
C5—H5···O4^iv^	0.950	2.500	3.445 (1)	175.0
C10—H10B···O1^x^	0.990	2.844	3.778 (1)	157.5
C8—H8B···Cg1^xi^	0.990	2.829	3.671 (1)	143.4

Symmetry codes: (i) −*x*+1, −*y*+1, −*z*−1; (iv) −*x*+2, −*y*, −*z*; (x) *x*, *y*, *z*+1; (xi) −*x*, −*y*, −*z*+1.
